# A Spatial Point Feature-Based Registration Method for Remote Sensing Images with Large Regional Variations

**DOI:** 10.3390/s25216608

**Published:** 2025-10-27

**Authors:** Yalun Zhao, Derong Chen, Jiulu Gong

**Affiliations:** School of Mechatronical Engineering, Beijing Institute of Technology, Beijing 100081, China; 3220205033@bit.edu.cn (Y.Z.); cdr@bit.edu.cn (D.C.)

**Keywords:** point feature, image registration, regional variation, remote sensing images, scale invariance, rotational invariance

## Abstract

The accurate registration of image pairs is an indispensable key step in the process of disaster assessment, environmental monitoring, and change detection. However, obtaining correct matches from input images is difficult, especially from images with significant resolution and regional variations. The current image-registration algorithms perform poorly in this application scenario. In this article, a spatial point feature-based registration method is proposed for remote sensing images with large regional variations. First, a new edge keypoint extraction method is designed that selects points with gradient magnitude maxima around the neighborhood of the edge line segments as keypoint features. Then, the feature descriptors for each keypoint are constructed based on the geometrical distribution (distance and orientation) of each keypoint. Considering the stability of the distribution of the edge contours, our constructed descriptor vectors can be well used for image pairs with large resolution and regional variations. In addition, all feature descriptors in this method are constructed and matched in the rotated image pyramid. Finally, the fast sampling consensus algorithm is applied to eliminate mismatches. In test images with various scales, rotation angles, and regional variations, the proposed method achieved pixel-level root mean square error, and the average registration precision is nearly 100%. Meanwhile, our proposed method’s rotation and scale invariance are verified by rotating and downsampling the image pairs extensively. In addition, compared with the comparison algorithms, the method proposed in this paper has better registration performance for images with resolution and regional variations.

## 1. Introduction

Owing to the convenience and intuitiveness characteristics of RS images, various reconnaissance means, such as reconnaissance aircraft, unmanned aerial vehicles (UAVs), and satellites, are widely used in modern battlefields to obtain enemy intelligence, monitor battlefield situations, and provide favorable support for combat decision making. By conducting change analysis on pre- and post-damage images, the damage effect on the target area can be rapidly assessed, and it can also be applied to post-disaster assessment [1]. The RS images’ accurate registration is a necessary condition for change detection [2,3]. The image registration’s main purpose is to geometrically align images of the same scene. With the development of RS technology, change analysis of registered image pairs can quickly obtain damage information of the target area, which can effectively improve the convenience of post-disaster assessment, damage effect assessment of the target area, and other work [4,5].

With the increasing diversity of RS image-acquisition methods, there may be various shooting perspectives, cloud cover, and image resolution differences among heterogeneous images from satellites or unmanned aerial vehicles. Meanwhile, disasters and wars can cause significant regional variations between the image pairs. All these pose significant challenges to the accurate registration of RS images and bring difficulties to the rapid progress of subsequent work such as change analysis and damage assessment. Over the years, many scholars have achieved certain research results in image registration. Most research regarding image registration concentrate on feature extraction, feature description, and feature matching. Traditional registration methods are generally divided into the following two types: region-based image-registration algorithms and feature-based image-registration algorithms.

Region-based image-registration methods, such as mutual information [6], calculate the same region’s correlation value in the image pair and maximize the correlation value to find the best matches. Such algorithms rely on local regional features of the image and are difficult to apply to image pairs with significant regional differences. Compared with region-based image-registration methods, the latter type of method is more widely used. As for the latter type of method, the point features [7,8,9,10,11,12,13,14,15,16,17,18] and line segment features [19,20,21,22,23,24,25,26,27,28,29,30,31,32] are the most commonly applied features. Among them, image-registration algorithms based on point features achieve registration by extracting keypoints in the images and establishing point-to-point correspondence. For example, the author of [7] first proposed the classic SIFT registration method in 2004. As the most famous and widely used point feature-based registration algorithm, this algorithm first extracts keypoints in the Gaussian pyramid. Then, it calculates the feature descriptor for matching by statistically analyzing the gradients of all pixels around the keypoint’s neighborhood region. This algorithm has good robustness and is invariant to rotation and scale. Subsequently, researchers improved and optimized the SIFT registration method and proposed other algorithms [8,9,10]. For instance, the algorithm UR-SIFT optimized the keypoints’ uneven distribution in the SIFT algorithm. Similarly, to obtain uniformly distributed keypoints, the author of [14] improved the ORB keypoint detector and proposed a new keypoint detection method. Then, the HOG [15] feature descriptor was constructed in the normalized image for matching. Additionally, the author of [16] combined the phase correlation-based method with the SIFT method and proposed a coarse-to-fine registration method for registration. In summary, the existing point feature-based registration methods are relatively dependent on local area features around the keypoints’ neighborhood regions and are, thus, difficult to be applied to image pairs with significant variations in resolution and regional areas.

Compared with point features, edge line segment features may contain spatial structure information and have better stability. Currently, some researchers have proposed some registration methods that are based on line segment features [19,20,21,22,23,24,25,26,27,28,29,30,31,32,33]. For example, the method in [19] used line segment features to replace keypoint features and constructed descriptors based on the distribution of gradient characteristics around the neighborhood of each line segment, which can be called MSLD. For urban area images, the authors of [21] proposed an image-registration method according to the intersection structure of line segments. This method first used the line descriptor MSLD for matching to obtain the initial matches and then eliminated incorrect matches using a graph-based outlier-removal strategy. For multi-temporal RS images, the authors of [23] first used the line descriptor MSLD for matching to obtain the initial matches. Then, the affine transformation parameters between the input image pair are estimated by constructing intersection triplets. For image pairs with significant background changes, the authors of [26] proposed a line feature-based registration method, which mainly utilized the distribution relationship of each line segment (relative positions and directions between each line segment) to construct descriptors for registration. In this method, the authors used each line segment’s midpoint to represent the precise position of a certain line segment and used the midpoints to calculate the transformation parameters. The authors of [27] proposed a rotation-invariant registration method using edge line features, which designed feature descriptors according to the relationship of each line segment for matching and achieved relatively accurate registration results. This method is mainly designed for multi-temporal images of the same source with the same resolution and performs poorly for images with significant resolution differences. The author of [28] constructed a new line-point invariant using the intersection point of coplanar lines for matching. The method in [34] performed image registration combining features such as line segments and keypoints. The author of [35] proposed a relatively novel keypoint extraction method. This method first detected line segments and then searched for points with gradient maxima in the neighborhood of the line segments as keypoints. Finally, the HOG feature descriptors are constructed using the regional features around the keypoint neighborhoods for matching. In general, the current line segment-based registration algorithms are relatively dependent on the regional and keypoint information in the neighborhood of line segments. All these make them difficult to be applied to remote sensing image pairs with significant variations in resolution and regional areas.

In addition, over the years, the techniques of deep learning have undergone extensive development and found widespread application in the image processing field, and some deep learning-based registration methods have been proposed [36,37,38,39,40,41]. These methods obtain a deep learning inference model suitable for specific application scenarios through iterative training using a vast amount of labeled data and then output relatively accurate registration results. However, because of the scarcity of extensive datasets, especially the absence of images with significant resolution and regional variations, deep learning methods’ application in the future is limited.

Summarizing the constraints of the existing registration methods, this paper takes image pairs with large resolution and regional variations as the research object and combines edge line segment features with keypoint features to establish feature descriptors for registration based on the consistency of the distribution of target positions and the stability of the main shape contours. This paper proposes a spatial point feature-based registration method for remote sensing images with large regional variations. The proposed method is based on the improvement method in [27] to further enhance the registration accuracy and achieve scale invariance. Compared to the method in [27], the main contributions and ideas of this paper can be summarized as follows: (1) an image pyramid is constructed to unify the scales of the reference image and the image to be registered; (2) the line segment features are combined with point features to construct feature descriptors and match them in the image pyramid to improve the registration precision; (3) the feature descriptors constructed in this paper can be well applied to image pairs with large resolution and regional variations; and (4) the method proposed in this paper has good rotation and scale invariance. In test images with different scales, rotation angles, and regional variations, the proposed method achieved a pixel-level registration root mean square error, and our method’s average registration precision is nearly 100%. Meanwhile, our proposed method’s rotation and scale invariance are verified by rotating and downsampling the image pairs extensively. In addition, compared with the comparison methods, the method proposed in this paper has better registration performance for images with resolution and regional variations.

The remaining is structured as follows: In Section 2, a detailed introduction to all steps of our method proposed in this article is provided. In Section 3, we first introduce our test images and evaluation indexes and then demonstrate our method‘s matching performance, scale invariance, rotation invariance, and its comparison with comparative algorithms. Section 4 discusses the keypoints and difficulties of our method. In Section 5, we summarize the proposed method and future research.

## 2. Methodology

**Figure 1:**
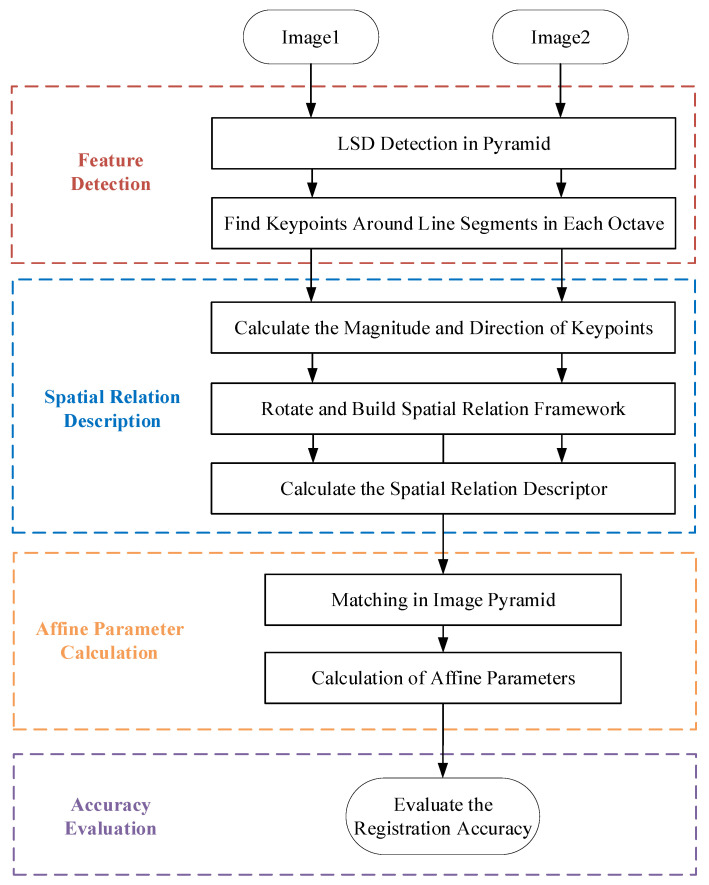
Framework of our proposed method.

The content of our proposed registration method mainly includes line segment detection, extraction of keypoints, descriptor calculation, matching, and affine transformation parameter calculation. We provide an introduction to our proposed method in this section. As shown in Figure 1, it presents the overall framework of our proposed registration method.

### 2.1. Feature Extraction

We respectively extract the line segment features and keypoint features from the input image pair. We consider that there may be significant scale and regional variations between the images to be registered, which may lead to the breakage or absence of corresponding line segment features in the image pairs to be registered. Therefore, to obtain more correct matches and improve the accuracy of the image registration, keypoint feature extraction was performed after the extraction of line segment features. Finally, the edge line segment features and the keypoint features are combined to construct descriptors. As shown in Figure 1, we provide detailed introductions to the feature extraction part in the following two sections: line segment extraction and keypoint extraction.

#### 2.1.1. Line Segment Extraction

Edge line segment features in images often contain rich semantic and spatial structure information. Nevertheless, for image pairs with significant resolution and regional variations, edge segments of the corresponding area may become fragmented or missing. Fragmented line segments can further affect the registration results. To overcome the fragmentation of edge line segments, an image pyramid is constructed to unify the scale of the input image pairs and extract edge line segment features and keypoint features within each octave of the image pyramid.

**Figure 2:**
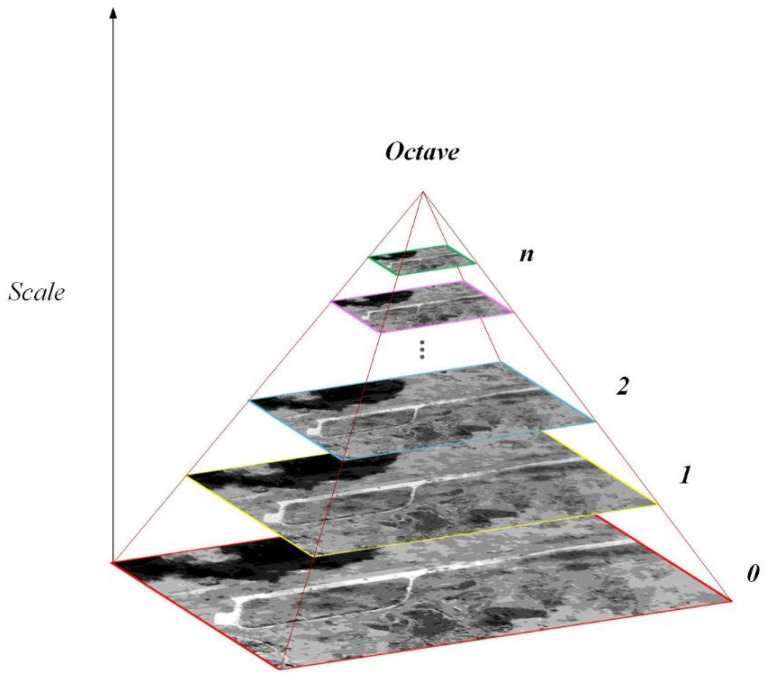
Feature extraction in an image pyramid.

As shown in Figure 2, we construct an image pyramid consisting of *n* octaves for the input images. We take the image at the bottom of the pyramid as the input image, and each of the remaining octaves are obtained by consecutive scale downsampling on the input image. Each octave contains only one layer of image, and, thus, we can obtain images with different resolutions through this image pyramid.

Considering that in images with different resolutions, the positions and quantities of the extracted line segments will vary significantly. For instance, in large-scale images, only a few edge line segments can be detected, and the positions of these segments are not very precise. In contrast to the formula $O=\left\lfloor \log_{2}\min\left(R,C\right)-2\right\rfloor$, adopted in the algorithm in [7], in order to obtain relatively sufficient line segments with precise positions on each octave in the pyramid, the number of octaves in the pyramid can be calculate through the following formula:

(1) $$O\;=\;\left\lfloor \log_{2}\min\left(R,C\right)-5\right\rfloor$$

where *R* indicates the input image’s rows, and *C* indicates the input image’s columns.

For the same reason, we choose $\sqrt{2}$ as the downsampling factor to build the image pyramid. For example, if the input image’s size is 1024 × 1024, it can be calculated that the image pyramid’s octave is 5. Then, the size of the image in the 5th octave is 256 × 256. In this way, for the large-scale image within the pyramid, a sufficient number of line segments can also be extracted for the subsequent construction of feature descriptors, while still maintaining good scale invariance.

**Figure 3:**
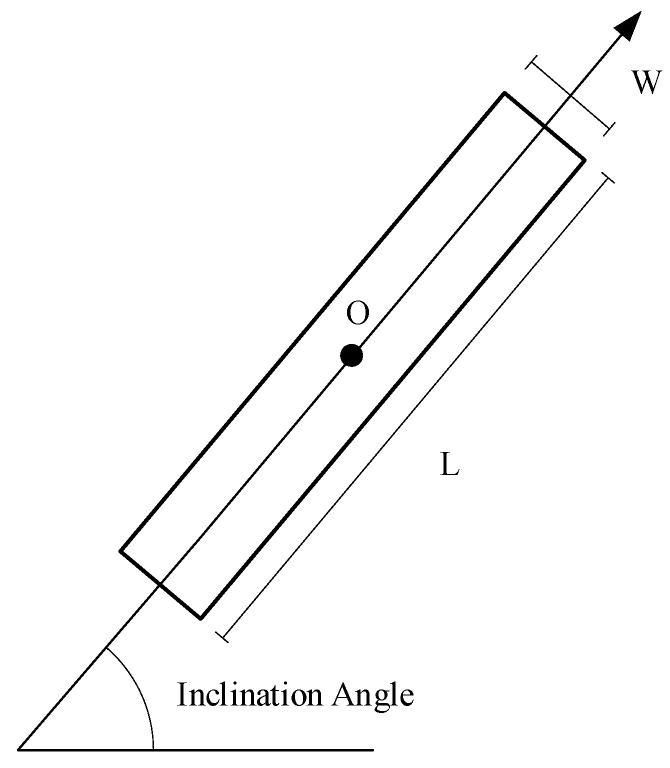
LSD line segment.

After the image pyramid is constructed, we first apply a Gaussian filter and bilateral filter to each octave image to reduce the influence of noise and obtain as many line segment features as possible [35]. Then, we use the LSD line detection algorithm proposed by the author of [42] to extract line features from each octave image. An LSD line segment can be shown in Figure 3.

Finally, the interfering line segments are eliminated by setting the corresponding length thresholds for each octave image within the image pyramid (considering the regional variations between image pairs, we set a small threshold to preserve more line segments). For the input image, we can obtain a set of line segments, *L*. This line segment set contains all the line segments in the images of each octave within the image pyramid. For each line segment detected in any octave image, it is represented by the inclination angle, length, and midpoint coordinates. The set of line segments, *L_s_*, extracted from any octave and the set of all line segments, *L,* of the input image can be, respectively, expressed as follows:

(2) $$\begin{array}{l} L_{s}=\left\{l_{si}:(p_{si},\theta_{si},e_{si}),i=1,2,\ldots n\right\}\\ L=\left\{L_{s},s=1,2,\ldots O\right\} \end{array}$$

where $p_{si}$ represents the line segment’s midpoint coordinate, $e_{si}$ represents the line segment’s length, $\theta_{si}$ represents the line segment’s inclination angle, *n* represents the line segments’ number in a certain octave, and *O* represents the octaves of image pyramid. The inclination angle’s interval is within [0~180°].

#### 2.1.2. Keypoint Extraction

As mentioned above, the number and positions of line segments detected in images with different scales may vary significantly, which can affect the accuracy of the subsequent registration. Therefore, we take the line segments set *L* as the input and search for the true edge points around each line segment as keypoint features. Then, we construct feature descriptors by using the spatial relationships (relative distances and directions) among each keypoints.

First, we calculate all of the pixels’ gradient directions and gradient magnitudes in each octave of the image pyramid constructed in Section 2.1.1. For the gradient direction at a given pixel point (r,c) in any octave image, we calculate it using the following formula:

(3) $$g_{x}(r,c)=\frac{v\left(r+1,c\right)+v\left(r+1,c+1\right)-v\left(r,c\right)-v\left(r,c+1\right)}{2}$$

(4) $$g_{y}(r,c)=\frac{v\left(r,c+1\right)+v\left(r+1,c+1\right)-v\left(r,c\right)-v\left(r+1,c\right)}{2}$$

where v(r,c) indicates the pixel point’s grayscale value, $g_{x}(r,c)$ indicates the gradient value along the x-direction at the point (r,c), and $g_{y}(r,c)$ indicates the gradient value along the y-direction at point (r,c).

The gradient magnitude of point (r,c) will be calculated using the following formula:

(5) $$G\left(r,c\right)=\sqrt{g_{x}^{2}\left(r,c\right)+g_{y}^{2}\left(r,c\right)}$$

The gradient direction of point (r,c) will be calculated using the following formula:

(6) $$\theta =\arctan\left(-\frac{g_{x}\left(r,c\right)}{g_{y}\left(r,c\right)}\right)$$

**Figure 4:**
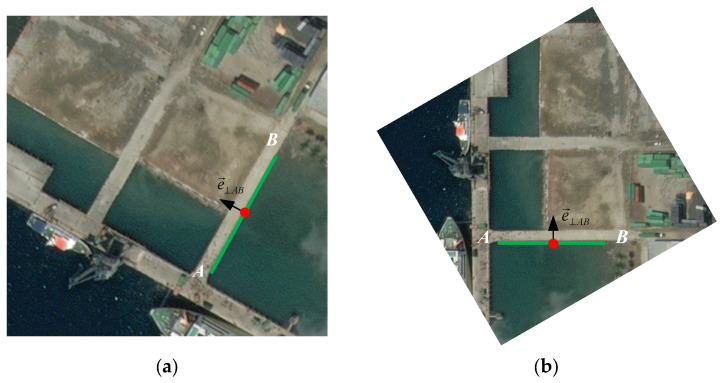
A real image pair: (**a**) an example of a line in an image before rotation; (**b**) an example of the line in a rotated image.

Then, we traverse the line set, *L_s_,* extracted from each octave, take the midpoint of any line segment, *l_si_*, in a certain octave image as the rotation center, and rotate the image by the inclination angle, *θ_si_*. We make the line segment, *l_si_*, horizontal in the rotated image. As shown in Figure 4, a pair of real images are presented (the state before and after rotation). Here, ${\vec{e}}_{⊥AB}$ represents the central normal vector perpendicular to the line segment *l_AB_* and pointing towards the highlighted side of the line segment *l_AB_*. We refer to the algorithm in [27] to calculate the normal vector’s direction and ensure that the line segment *l_AB_* in the rotated image is in a horizontal position with the central normal vector, ${\vec{e}}_{⊥AB}$, pointing upwards. The normal vector, ${\vec{e}}_{⊥AB}$, can be expressed as follows [43]:

(7) $${\vec{e}}_{⊥AB}=\left(\frac{\left(y_{a}-y_{b}\right)}{\sqrt{{\left(y_{a}-y_{b}\right)}^{2}+{\left(x_{b}-x_{a}\right)}^{2}}},\frac{\left(x_{b}-x_{a}\right)}{\sqrt{{\left(y_{a}-y_{b}\right)}^{2}+{\left(x_{b}-x_{a}\right)}^{2}}}\right)$$

where $\left(x_{a},y_{a}\right)$ and $\left(x_{b},y_{b}\right)$ represent the coordinates of pixel points *A* and *B* in image coordinate system, respectively.

**Figure 5:**
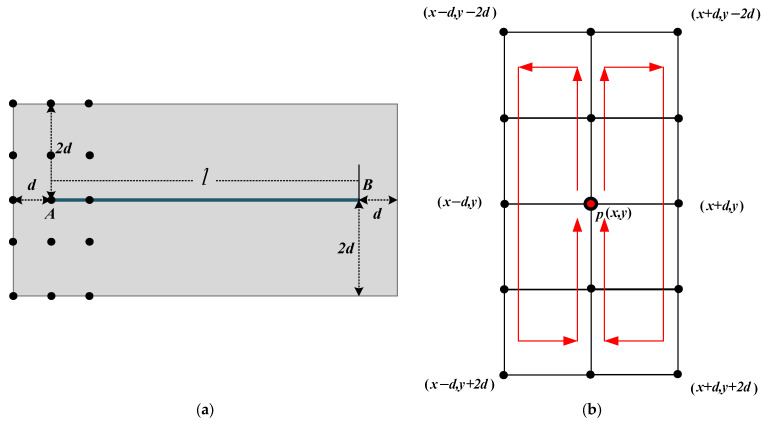
Keypoint search around the line segment’s neighborhood: (**a**) illustration of the search area around the neighborhood of a line segment; (**b**) illustration of the keypoint search around a line segment’s neighborhood.

Finally, for *l_AB_* with the horizontal position after rotation, we extend d pixels along the horizontal direction at both the end of the line segment and hte extend 2d pixels along the perpendicular direction on both sides of the line segment to construct a rectangular area (as shown in Figure 5a). For the search for keypoints around the neighborhood of the horizontal line segment, *l_AB_*, we first start from the endpoints and select the central pixel point at an interval of *2d*($p_{o}\left(x,y\right),\;o=1:2d:e_{AB}$). Taking the pixel point *p_o_* as the center, we select $\left(2d+1\right)\times \left(4d+1\right)$ candidate points within a rectangular area with a width of *2d* and a height of *4d*. Then, the set of pixel points in the rectangular area can be expressed as $\left\{\left(x-d,y-2d\right),\left(x,y-2d\right),\ldots \left(x,y\right),\ldots \left(x,y+2d\right),\left(x+d,y+2d\right)\right\}$. Compare the gradient magnitudes of all candidate points within the rectangular area, and search for the points with maximum gradient amplitude as keypoints in the neighborhood around the central pixel point, *p_o_*. After obtaining the keypoints, we convert them to the original image through the inverse rotation matrix.

(8) $$\begin{array}{l} G\left(p_{k}\right)=Max\{G\left(x-d,y-2d\right),G\left(x,y-2d\right),\ldots G\left(x,y\right)\},\\ \left.\;\;\;\;\;\;\;\;\;\;\;\;\ldots G\left(x,y+2d\right),G\left(x+d,y+2d\right)\right\} \end{array}$$

where *G* indicates the magnitude of the gradient at a certain point.

As described in Section 2.1.1 above, in any octave image, a set of line segments, $L_{s}=\left\{l_{si}:(p_{si},\theta_{si},e_{si}),i=1,2,\ldots n\right\}$, can be extracted, where $e_{si}$ represents the length of *l_si_* and *n* indicates the number of line segments in the set. Therefore, for any input image, the number of all keypoints can be expressed as follows:

(9) $$N_{total}=\sum_{s=1}^{O}\sum_{i=1}^{n}floor(e_{si}/(2d+1))$$

where *n* represents the number of line segments extracted from an octave image in the image pyramid; *O* indicates the number of octaves in the image pyramid; *d* indicates the length of a line segment extending toward the line segment’s end. We set the parameter *d* to 1 in this article.

### 2.2. Descriptor Construction

After the above steps, we obtain two keypoint sets in the input image pair reference image and the image to be registered, which can be represented as $P=\left\{p_{sj}\right\}(s=1,2,\ldots O;j=1,2,\ldots m)$ and $P^{\prime }=\left\{{p^{\prime }}_{sj}\right\}(s=1,2,\ldots O^{\prime };j=1,2,\ldots m^{\prime })$. Here, *s* represents the index of the octave image in which the current keypoint is located, and *j* indicates the index of the current keypoint in a certain octave image. Our goal is to construct feature descriptors in the octave images corresponding to each keypoint to find the best matches between keypoints $p_{sj}$ and ${p^{\prime }}_{sj}$. Considering the resolution and regional variations between the image pairs, the length, position, and regional features around the line segments at the same position in the image pairs have significant differences. Therefore, we construct feature descriptors based on the stability of the relative position distribution (distance and direction) between edge keypoints. In our method, each keypoint, $p_{sj}(x,y)$, extracted in Section 2.1 is associated with the line segment, $l_{si}$, it depends on. For example, when constructing the feature descriptor for a certain keypoint, $p_{sj}(x,y)$, we first rotate the image by the angle *θ_si_* and then calculate the feature descriptor using the relative position relationship between the remaining keypoints and $p_{sj}(x,y)$ in the rotated image to ensure the rotational invariance of the method proposed in this paper. For any keypoint, $p_{sj}$, in a certain octave image, we represent the relative position relationship between it and the other keypoints as follows:

(10) $$h_{sj}\left(k\right)=\left\{\left(p_{sj}-p_{so}\right)\in bin\left(k\right),\left(o=1,2,\ldots m,j\neq o\right)\right\}$$

where *bin* indicates the sectors in the histogram space; *k* indicates the index of the sectors in the histogram space; $\left(p_{sj}-p_{so}\right)$ indicates the spatial relationship between the keypoints $p_{sj}$ and $p_{so}$; and *m* indicates the remaining total number of keypoints, except for keypoint $p_{sj}$.

**Figure 6:**
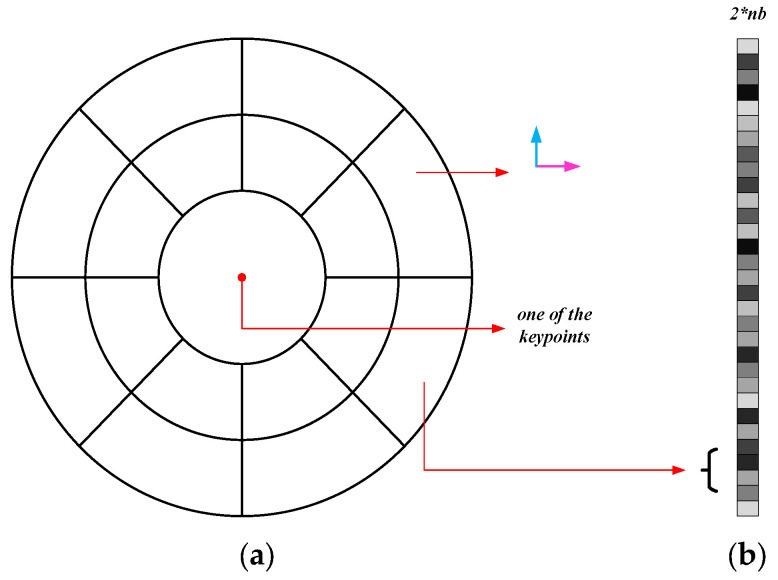
The feature descriptor’s histogram space: (**a**) histogram space; (**b**) feature descriptor.

In the proposed method, we construct a spatial circular ring structure to represent the spatial positional relationship among the keypoints. As shown in Figure 6a, we use concentric and equally radiused circular ring structures to construct a histogram space of the feature descriptors. Then, we calculate a vector with a length of 2×nb for each keypoint to describe it, which can be seen in Figure 6b. Here, $nb=ns\times \left(nr-1\right)+1$, *ns* indicates the number of sectors within a ring, *nr* represents the amount of circular rings distributed radially, and *nb* indicates the total amount of sectors. When calculating feature descriptors, we divide the gradient directions of all extracted keypoints into the x-direction and the y-direction. Within each small sector region, we accumulate the gradient magnitudes of all of the keypoints located in that region to calculate the histogram vector. The descriptor vector can be calculated as follows:

(11) $$\left\{\begin{array}{l} {h_{sj}}^{x}\left(k\right)=\sum_{x-direction}G_{x}\left(p_{sj}\right)\;\\ {h_{sj}}^{y}\left(k\right)=\sum_{y-direction}G_{y}\left(p_{sj}\right) \end{array}\right.,p_{sj}\in bin(k)$$

where $G_{x}\left(p_{sj}\right)$ represents the gradient magnitude component of the keypoint $p_{sj}$ along the x-direction, and $G_{y}\left(p_{sj}\right)$ represents the gradient magnitude component of $p_{sj}$ along the y-direction.

Finally, the feature descriptor vector of the keypoint in a certain octave image of the image pyramid can be represented as follows:

(12) $$h_{sj}\left(o\right)=\left\{{h_{sj}}^{x}\left(1\right),{h_{sj}}^{y}\left(1\right),{h_{sj}}^{x}\left(2\right),{h_{sj}}^{y}\left(2\right),\ldots {h_{sj}}^{x}\left(nb\right),{h_{sj}}^{y}\left(nb\right)\right\}$$

### 2.3. Feature Matching

After the above operations, we can obtain a set of feature descriptor vectors in input images. Here, we denote the keypoints in an image pair to be registered as $p_{sj}$ and ${p^{\prime }}_{sj}$ and the corresponding descriptors as $h_{sj}\left(o\right)$ and ${h_{sj}}^{\prime }\left(o\right)$, respectively. In this paper, the NNDR method adopted by the author of [7] is adopted to calculate the similarity of descriptors $h_{sj}(o)$ and ${h_{sj}}^{\prime }(o)$ in the reference image and the image to be registered. The distance of the keypoints can be calculated as follows:

(13) $$Dist=\sqrt{\sum_{o=1}^{2nb}{\left(h_{sj}(o)-{h_{sj}}^{\prime }(o)\right)}^{2}}$$

where $h_{sj}(o)$ and ${h_{sj}}^{\prime }(o)$ represents the vector of descriptor at keypoints $p_{sj}$ and ${p^{\prime }}_{sj}$. The length of the vector is 2×nb.

Finally, the FSC method [44] was employed to perform the operation of outlier removal and take the matches satisfying the error constraints as the final correct matches. In this paper, we consider the matching pairs with a real transformation error less than 3 pixels [14] after affine transformation as the final correct matches. Lastly, based on the matching results, we evaluated the registration accuracy.

## 3. Results

The performance of the proposed method will be evaluated by typical RS image pairs with significant regional variations. Additionally, to more comprehensively demonstrate our method’s effectiveness, we will apply it along with comparison algorithms such as method [7], method [14], method [26], and Method [27] to test images with different rotation angles and different downsampling scales for comparative experiments. In this paper, all the experiments were performed in MATLAB 2021b under the Windows 10 operating system, and the configuration of the computer was Intel Core I7-8750H CPU @2.20 GHz 16 GB RAM. Finally, we will provide a detailed introduction to test images, evaluation indicators, and registration results adopted in this paper.

### 3.1. Datasets

**Figure 7:**
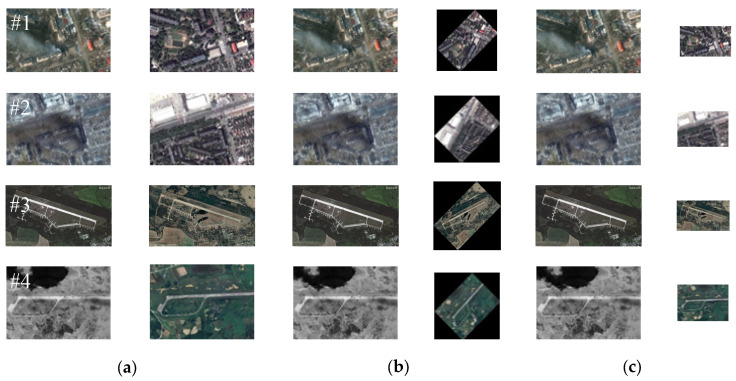
Examples of images of war damage: (**a**) original image pairs #1–4: image pair 1 (Mariupol), image pair 2 (Mariupol), image pair 3 (Saky Air Base, Russia), and image pair 4 (Ponikve Airport, Serbia); (**b**) image pairs with a 45° rotation; (**c**) image pairs with 2× downsampling.

**Figure 8:**
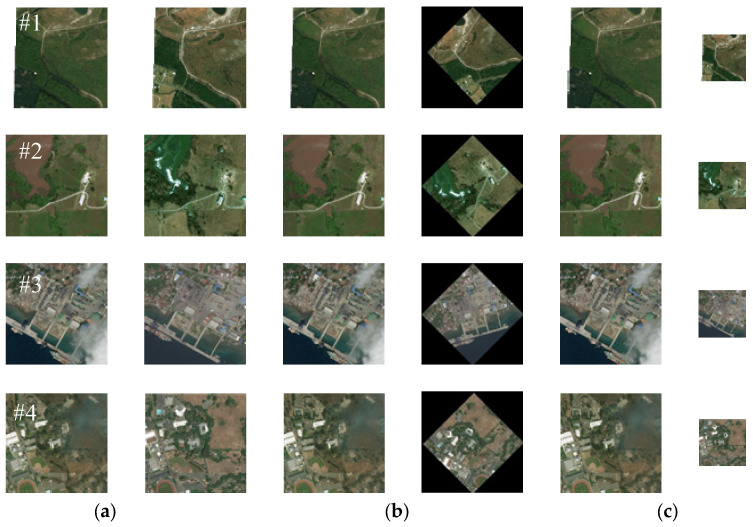
Examples of images of natural disaster damage: (**a**) original image pairs; (**b**) image pairs with a 45° rotation; (**c**) image pairs with 2× downsampling.

Our proposed registration method mainly focuses on image pairs with various angle and scale differences. This method also takes into account the significant regional variations between the pre- and post-damage image pairs. Therefore, referencing the method in [27], we select remote sensing image pairs before and after damage caused by wars and those caused by natural disasters to form the test dataset and conduct experimental verification of our proposed method’s effectiveness. Among them, the images after war damage are obtained from the Internet (e.g., Russia–Ukraine war), while the corresponding pre-damage images are downloaded from Google Earth. The image pairs before and after disaster damage are all selected from publicly available datasets [45]. Meanwhile, to comprehensively evaluate the proposed method’s effectiveness, we also performed multi-angle rotation and multi-scale downsampling operations on the test images. Figure 7 and Figure 8, respectively, show RS images before and after destruction caused by wars and natural disasters used in this paper. Table 1 presents the information for all of the test images adopted in this paper. Additionally, the rotated images shown in Figure 7b and Figure 8b did not undergo downsampling operations.

The test images used in the following experiments not only consider the smoke coverage, regional variations, and cloud occlusion resulting from war or disaster damage (as shown in Figure 7a and Figure 8a) but also consider various complex application scenarios such as possible angle and scale differences between heterogeneous images (as shown in Figure 7b,c and Figure 8b,c). Meanwhile, to further verify our method’s rotation and scale invariance, multi-angle rotation and multi-scale downsampling operations are conducted on the test images. As shown in Figure 7b and Figure 8b, the test image pairs are rotated by 45 degrees. Figure 7c and Figure 8c show the test image pairs that are downsampled by a factor of 2.

### 3.2. Evaluation Criterion

In the following text, we qualitatively compare the matching performance of our proposed method and the other existing algorithms by enlarging sub-images of the correct matching images. In addition, we quantitatively compare the registration performance of the proposed method and the other existing methods through indicators, such as the root mean square error (RMSE), precision, and the number of correct matches (NCM). All evaluation indicators are as follows:

(14) $$RMSE=\sqrt{\frac{1}{N_{ncm}}\sum_{j=1}^{N_{ncm}}\left({\left({x_{j}}^{r}-({x_{j}}^{m}{)}^{\prime }\right)}^{2}+{\left({y_{j}}^{r}-({y_{j}}^{m}{)}^{\prime }\right)}^{2}\right)}$$

(15) $$Precision=\frac{TP}{TP+FP}$$

(16) NCM=TP

where $N_{ncm}$ indicates the amount of correct matches, $\left({x_{j}}^{r},{y_{j}}^{r}\right)$ represents a pixel point in the reference image, $\left(({x_{j}}^{m}{)}^{\prime },({y_{j}}^{m}{)}^{\prime }\right)$ represents the point $\left({x_{j}}^{r},{y_{j}}^{r}\right)$ converted to the image to be registered, *TP* indicates the amount of correct matches in the final result, and *FP* indicates the amount of false matches in the final result. We set the RMSE of the mismatched image pair to 20 pixels.

### 3.3. Experimental Results

#### 3.3.1. Experimental Results Applied to Dataset 1

**Figure 9:**
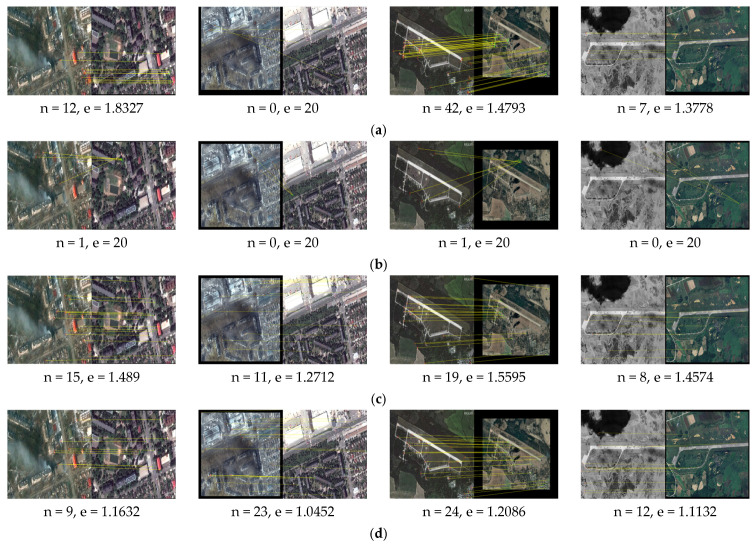

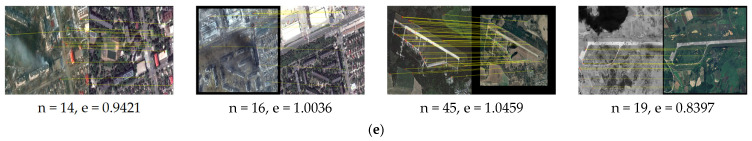
Matching results for images before and after war damage (the same scale, 0° rotation): (**a**) registration results for the algorithm in [7]; (**b**) registration results for the algorithm in [14]; (**c**) registration results for the algorithm in [26]; (**d**) registration results for the method in [27]; (**e**) registration results for the algorithm proposed in this paper.

**Figure 10:**
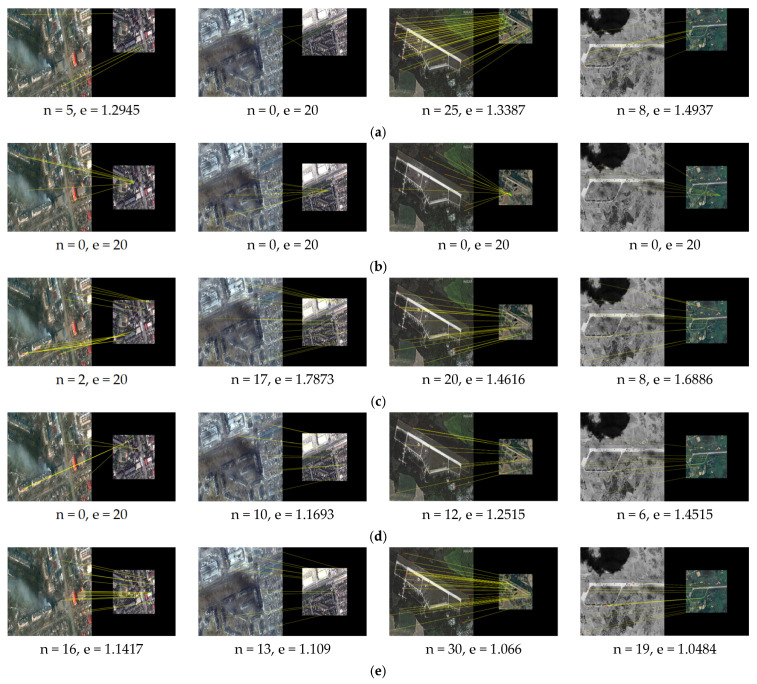
Matching results for images before and after war damage (2× downsampling, 0° rotation): (**a**) registration results for the algorithm in [7]; (**b**) registration results for the algorithm in [14]; (**c**) registration results for the algorithm in [26]; (**d**) registration results for the method in [27]; (**e**) registration results for the algorithm proposed in this paper.

**Figure 11:**
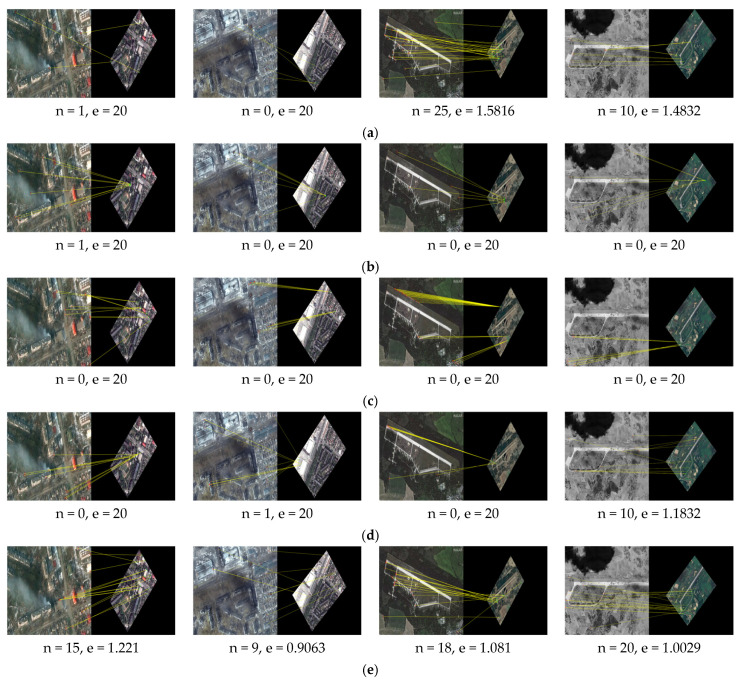
Matching results for images before and after war damage (2× downsampling and 45° rotation): (**a**) registration results for the algorithm in [7]; (**b**) registration results for the algorithm in [14]; (**c**) registration results for the algorithm in [26]; (**d**) registration results for the method in [27]; (**e**) registration results for the proposed algorithm.

We apply our proposed method and the comparison methods SIFT [7], LNIFT [14], RMSLM [26], and the method in [27] to Dataset 1 for comparative experiments. Figure 9, Figure 10 and Figure 11, respectively, show our proposed method’s results and the representative methods’ results on Dataset 1 in multiple application scenarios, such as the same scale without rotation, 2× downsampling without rotation, and 2× downsampling with a 45° rotation. In Dataset 1, the images of war damage have different degrees of damage (for example, the image pair #2’s damage degree is relatively large, and in the post-damage image, the buildings are severely damaged and have large areas of burn marks. The image pair #3’s damage degree is the smallest, and the regional variations between the image pair are relatively small). Thus, the image pairs can well reflect the comparison of the registration method proposed in this paper and the comparison methods. In addition, considering that the method proposed in this article sacrifices operational efficiency by increasing the number of keypoints involved in the calculation to solve the problem related to difficulty in achieving accurate registration between images with large regional and scale differences, we do not calculate or compare the algorithm’s runtime.

As shown in Figure 9, the algorithm SIFT [7] fails to register image pair #2 with significant regional variations but achieves correct registration on image pairs #1, #3, and #4 with small regional variations, though with considerable registration errors. As illustrated in Figure 10 and Figure 11, after downsampling the pre-damage image by a factor of two and rotating it by 45°, the algorithm SIFT still maintains a relatively good registration effect. This indicates that the algorithm SIFT has good rotational and scale invariance, but this method cannot correctly register image pairs with significant regional differences. The algorithm [14] fails to register all test images in scenarios of the same scale, multi-scale differences, and rotation, suggesting that this algorithm has poor scale and rotational invariance and cannot be applied to image pairs with large regional variations. The algorithm RMSLM [26] can achieve relatively accurate registration for all test images at the same scale. However, after rotating the pre-damage image by 45°, the algorithm cannot correctly register all test image pairs, which suggests that the method RMSLM can be well applied to images with significant regional differences but lacks rotation invariance.

As shown in Figure 9, the algorithm in [27] registers all of the test images correctly. As shown in Figure 10 and Figure 11, after downsampling the pre-damage image by a factor of two, the registration fails for image pair #1. After downsampling the pre-damage image by a factor of two and rotating it by 45°, the algorithm only correctly registers pair #4, which indicates that the method in [27] has poor scale invariance and basically loses rotational invariance when there are scale differences between image pairs. In summary, the proposed registration algorithm can successfully match all of the test images in multiple scenarios with different angles and scales, and the registration effect is superior to other comparison algorithms. The proposed algorithm has good scale and rotation invariance.

As shown in Table 2 below, the proposed algorithm and comparison methods’ quantitative matching results applied to Dataset 1 are presented. As indicated in Table 2, in all application scenarios, our proposed algorithm achieves the highest number of matching pairs and matching accuracy. At the same time, the proposed method has the smallest registration error. In conclusion, the proposed algorithm has good scale and rotation invariance.

**Figure 12:**
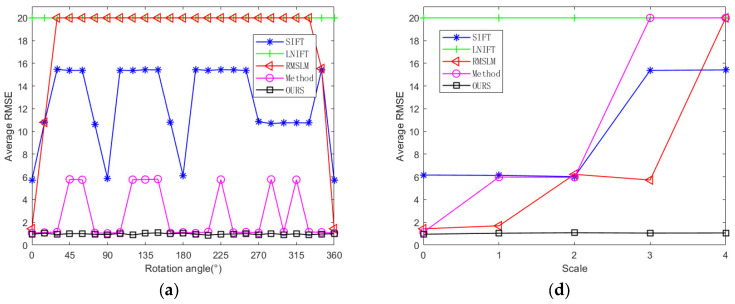

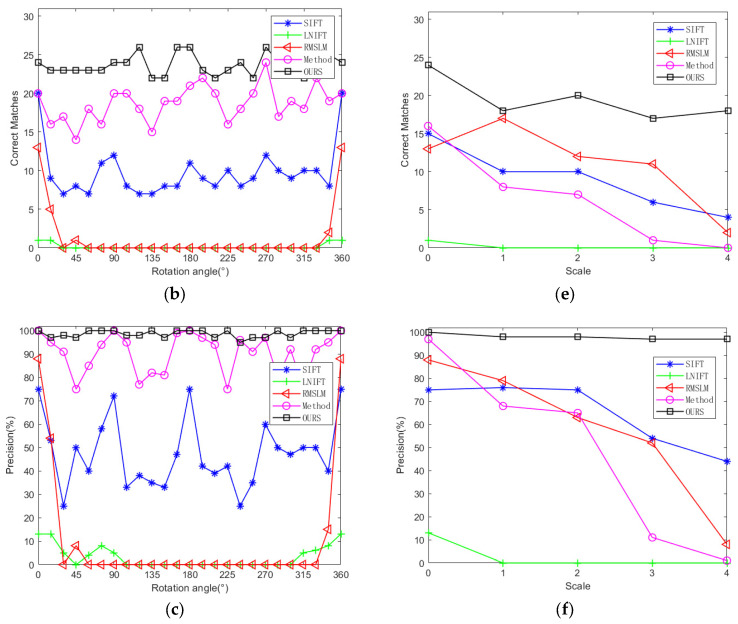
Quantitative registration results of algorithms SIFT [7], LNIFT [14], RMSLM [26], Method [27] and Ours for the images of war damage: (**a**) comparison of the average RMSE with the change of angle; (**b**) comparison of NCM with the change of angle; (**c**) comparison of the average precision with the change of angle; (**d**) comparison of the average RMSE with the change of scale; (**e**) comparison of NCM with the change of scale; (**f**) comparison of the average precision with the change of scale.

To further verify the proposed algorithm’s rotation and scale invariance and to make a fair comparison with other methods, we rotated the test images with an interval of 15° at the same scale to conduct the rotation invariance comparative experiments. We also performed multi-scale downsampling on the test images with the same rotation angle to conduct the scale invariance comparative experiments. As shown in Figure 12, the changes in the average registration accuracy, registration error RMSE, and NCM of our proposed algorithm and the comparison algorithms applied to Dataset 1 under different rotation angles and different downsampling scales are shown. As illustrated in Figure 12, the average registration precision and NCM of our proposed algorithm are higher than those of the comparison algorithms, and the average registration error is smaller than those of the comparison algorithms. Among the comparison algorithms, the method in [27] performs the best and has good rotation invariance. However, when there are scale differences between the image pairs, the method performs poorly and there is a lack of scale invariance. Overall, the proposed method has the best registration performance and stable rotation and scale invariance at the same time.

#### 3.3.2. Experimental Results Applied to Dataset 2

**Figure 13:**
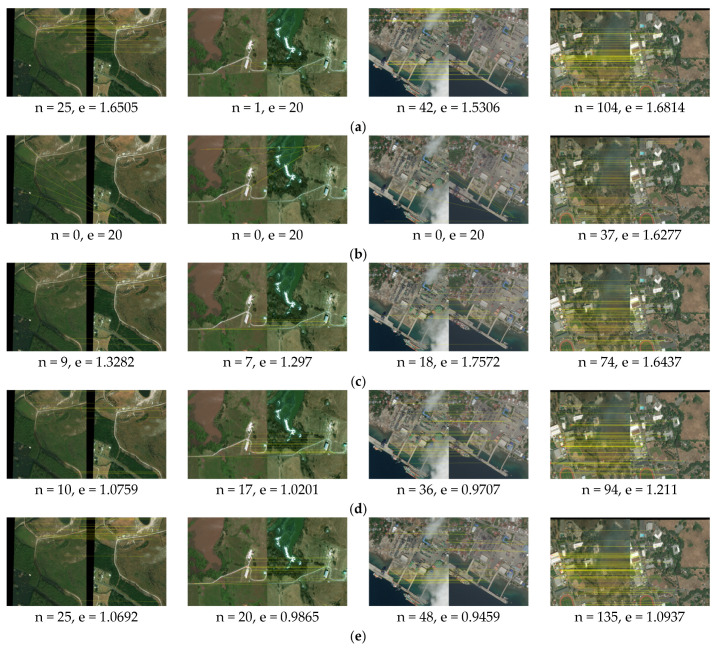
Registration results of the images of disaster damage (the same scale, 0° rotation): (**a**) registration results for the algorithm in [7]; (**b**) registration results for the algorithm in [14]; (**c**) registration results for the algorithm in [26]; (**d**) registration results for the method in [27]; (**e**) registration results for the algorithm proposed in this paper.

**Figure 14:**
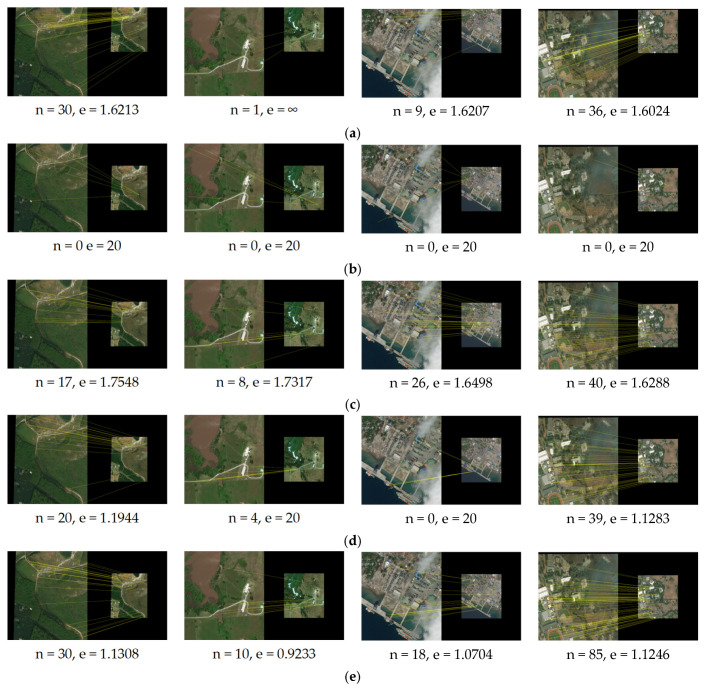
Registration results of the images of disaster damage (2× downsampling, 0° rotation): (**a**) registration results for the algorithm in [7]; (**b**) registration results for the algorithm in [14]; (**c**) registration results for the algorithm in [26]; (**d**) registration results for the method in [27]; (**e**) registration results for the algorithm proposed in this paper.

**Figure 15:**
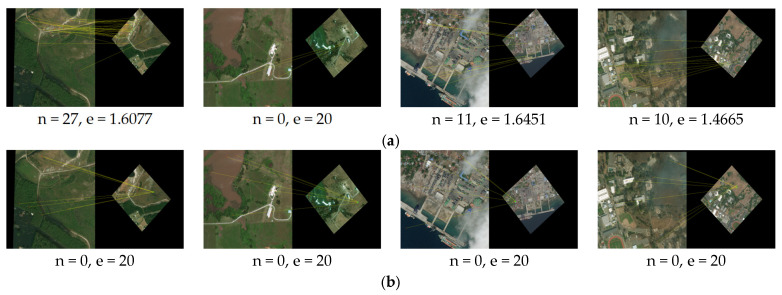

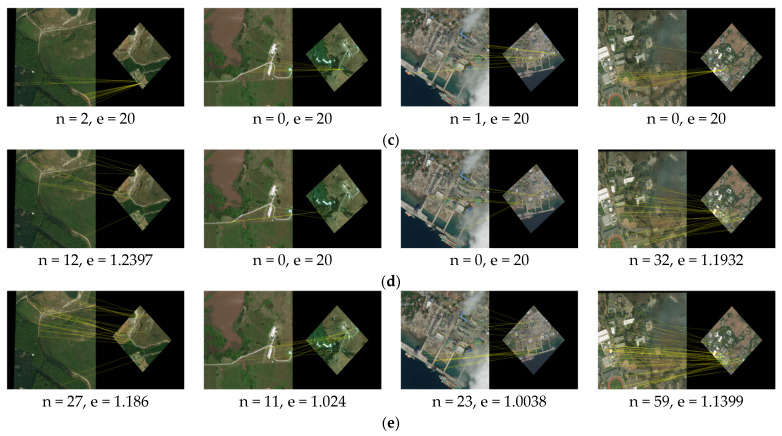
Registration results of the image of disaster damage (2× downsampling, 45° rotation): (**a**) registration results for the algorithm in [7]; (**b**) registration results for the algorithm in [14]; (**c**) registration results for the algorithm in [26]; (**d**) registration results for the method in [27]; (**e**) registration results for the proposed algorithm.

We apply our method proposed in this paper and the other methods SIFT [7], LNIFT [14], RMSLM [26], and the method in [27] to Dataset 2 for comparative experiments. Figure 13, Figure 14 and Figure 15, respectively, show the results of our algorithm and the representative algorithms on Dataset 2 in multiple application scenarios, such as the same scale without rotation, 2× downsampling without rotation, and 2× downsampling with 45° rotation. In Dataset 2, the test images simultaneously contain image pairs before and after damage in various disaster scenarios, such as hurricanes, floods, tsunamis, and wildfires. Moreover, the image pairs have different degrees of damage and, thus, can well reflect the performance comparison of our method and the representative methods. In addition, considering that the method proposed in this article sacrifices operational efficiency by increasing the number of keypoints involved in the calculation to solve the problem of difficult accurate registration between images with large regional and scale differences, we will not calculate or compare the algorithm’s runtime.

As can be seen from Figure 13, the algorithm in [7] failed to register image pair #6 with significant regional variations but was successful for the rest of the image pairs. The algorithm LNIFT [14] failed to register all of the test images. The algorithm RMSLM [26], the algorithm in [27], and our proposed method all achieved relatively accurate registration for all of the test image pairs. However, our proposed method had the smallest registration error, the highest registration precision, and the largest NCM. As shown in Figure 14 and Figure 15, after downsampling the pre-damage image by a factor of two and rotating it by 45°, algorithms SIFT [7] and LNIFT [14] maintained the original registration effect for all test image pairs. Algorithm RMSLM [26] only completed the registration in the 2× downsampling scenario and failed to register all test image pairs in the 45° rotation scenario. In addition, in both the 2× downsampling and 45° rotation scenarios, the algorithm in [27] only achieved correct registration for image pairs #5 and #8. In contrast, our registration method accurately registered all test images in scenarios with different scales and rotation angles, and the registration effect was superior to that of other comparison algorithms. In summary, the algorithm SIFT [7] has good rotational and scale invariance, but this method cannot correctly register images with significant regional variations. The algorithm RMSLM can be well applied to scenarios with significant regional variations but lacks rotational invariance. The method in [27] has poor scale invariance, and when there are scale differences between image pairs, this algorithm cannot maintain stable rotation invariance. In contrast, the proposed method has stable rotation and scale invariance.

As shown in Table 3 below, the quantitative registration results applied to Dataset 2 are presented. As indicated in Table 3, in all application scenarios, the proposed method has the highest number of matching pairs and matching accuracy. At the same time, the proposed method has the smallest registration error. In conclusion, the proposed algorithm has good scale and rotation invariance.

To further verify the proposed algorithm’s rotation and scale invariance and to ensure the fairness of the comparative experiments, we rotated the pre-disaster test images in Dataset 2 with an interval of 15° at the same scale to conduct the rotation invariance comparative experiments. In addition, we performed multi-scale downsampling on the pre-disaster test images at the same rotation angle to conduct the scale invariance comparative experiments.

**Figure 16:**
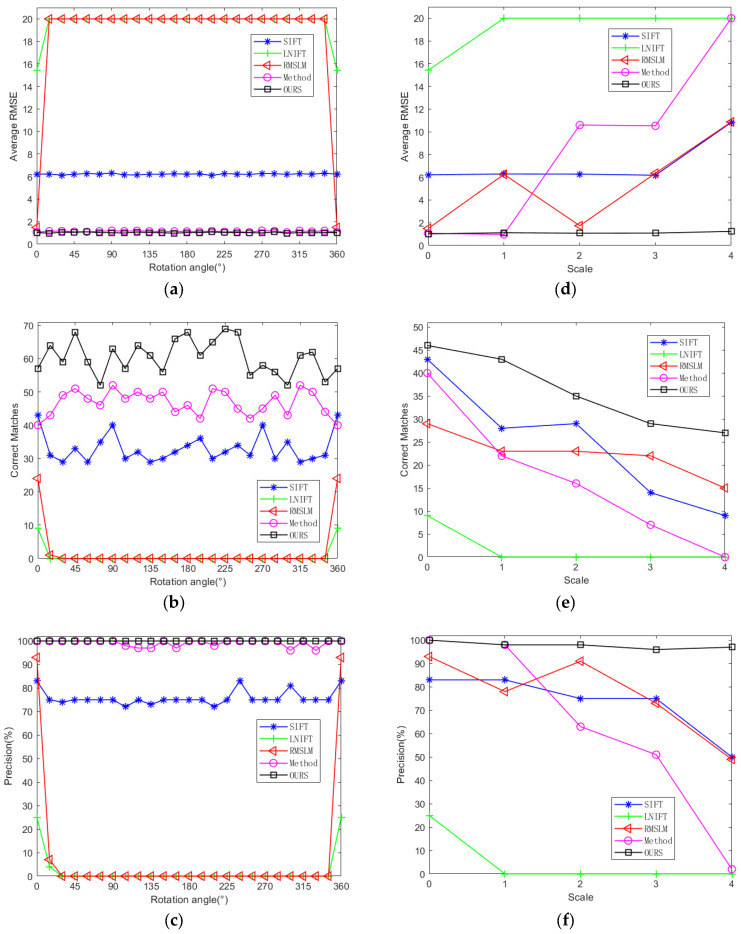
Quantitative registration results of algorithms SIFT [7], LNIFT [14], RMSLM [26], Method [27] and Ours for the images of disaster damage: (**a**) comparison of the average RMSE with the change of angle; (**b**) comparison of NCM with the change of angle; (**c**) comparison of the average precision with the change of angle; (**d**) comparison of the average RMSE with the change of scale; (**e**) comparison of the NCM with the change of scale; (**f**) comparison of the average precision with the change of scale.

As shown in Figure 16, it presents the changes in the average registration precision, registration error RMSE, and NCM of our proposed algorithm and the comparative algorithms applied to Dataset 2 under different rotation angles and different sampling scales. As can be seen from Figure 16, the registration precision and the number of correct matches of our proposed algorithm are higher than those of other comparative methods, and the average registration error is smaller than those of other comparative algorithms. Among the comparative methods, the algorithm SIFT [7] and algorithm in [27] maintain relatively stable registration effects at different rotation angles, demonstrating good rotation invariance. However, when there are scale differences between image pairs, they perform poorly, showing poor scale invariance. The algorithm RMSLM [26] only completed the registration when the test images were not rotated, which indicates that the algorithm RMSLM lacks rotation invariance. Overall, the proposed method in this paper has the best registration performance and stable rotation and scale invariance.

**Figure 17:**
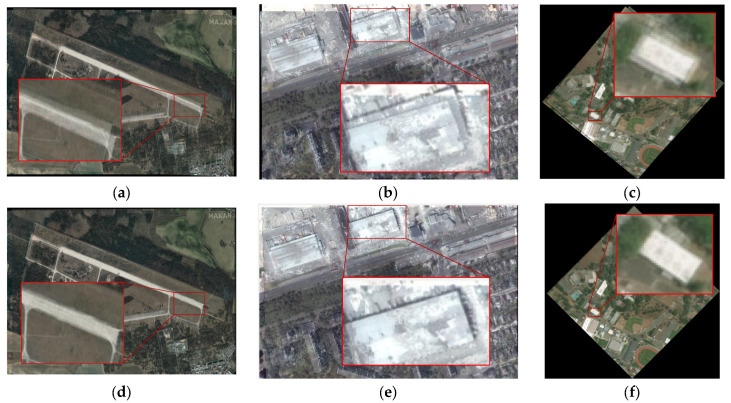
The final registration results of the proposed method and the comparison algorithms applied to the images #2, #3 and #8: (**a**) registration results of the algorithm RMSLM [26] applied to image pair #3 (the same scale, 0° rotation); (**b**) registration results of the method in [27] applied to image pair #2 (2× downsampling, 0° rotation); (**c**) registration results of the algorithm SIFT [7] applied to image pair #8 (2× downsampling, 45° rotation); (**d**) registration results of our proposed method applied to image pair #3 (the same scale, 0° rotation); (**e**) registration results of our method applied to image pair #2 (2× downsampling, 0° rotation); (**f**) the proposed method’s registration results applied to image pair #8 (2× downsampling, 45° rotation).

Finally, considering the advantages of each comparison algorithm, we, respectively, selected test image pairs #2, #3, and #8 to qualitatively compare the matching effects of the proposed algorithm with the comparison algorithms. As mentioned above, both the algorithm RMSLM [26] and algorithm in [27] can be more suitable for RS image pairs with large regional differences and have certain scale invariance. The algorithm SIFT [7] has good scale and rotation invariance, but it is difficult to apply to image pairs with large regional variations. Therefore, we applied the proposed method and the algorithm RMSLM [26] to image pair #3 (the same scale and 0° rotation) to qualitatively compare the registration results. We applied the proposed method and comparison algorithm in [27] to image pair #2 (2× downsampling and 0° rotation) to qualitatively compare the registration results. We apply the proposed method and comparison algorithm SIFT [7] to image pair #8 (2× downsampling and 45° rotation) with smaller regional differences to qualitatively compare the registration results. As shown in Figure 17, the matching results of images #2, #3, and #8 are presented, and the local regions are magnified to compare the registration effects. As can be seen from Figure 17, compared with representative methods, the proposed method has the best registration effect in scenarios with different scales and rotation angles.

## 4. Discussion

There may be phenomena, such as cloud cover, differences in shooting angles, and image resolutions, between heterogeneous image pairs from platforms like satellites and unmanned aerial vehicles. Meanwhile, the occurrence of disasters and wars can cause significant regional variations between the image pair. All these take significant challenges to the accurate matching of image pairs. This paper proposes a spatial point feature-based registration method for remote sensing images with significant resolution and regional differences. In test images with different scales, rotation angles, and regional differences, the proposed method achieved a pixel-level registration root mean square error, and our method’s average registration precision is nearly 100%. In addition, the comparison experiment results show that the proposed algorithm has a better matching performance for RS images with resolution and regional variations. Among the comparison algorithms, the algorithm RMSLM [26] can be well applied to RS images with significant variations. However, because this algorithm only calculates the affine transformation parameters through the midpoints of line segments, the NCM is small and the registration error is large. Moreover, this algorithm lacks scale and rotational invariance. The method in [27] can also be well applied to remote sensing image pairs with large differences and achieves rotation invariance. However, when there are scale differences between image pairs, this algorithm cannot achieve correct registration and lacks scale invariance. In addition, when there are scale and regional differences between image pairs, there may be positional deviations in the true edge segments of the same region, which will affect the registration effect of the line-based method [27]. In contrast, our proposed method designed a new edge keypoint extraction method that selects points with gradient magnitude maxima around the neighborhood of the edge line segments as keypoint features, increasing the NCM and reducing the registration error. Of course, due to the significant increase in the keypoint features involved in the calculation, the method proposed in this paper will have a longer running time. Because the method proposed in this article aims to solve the problem of difficult accurate registration between image pairs with large regional and scale differences, the efficiency of the algorithm has not been considered at present. Meanwhile, the proposed method builds feature descriptor vectors for all keypoints in the rotated image based on the geometrical distribution(distance and orientation) between each keypoint and performs matching within the image pyramid, achieving rotation and scale invariance of the proposed method.

Overall, our proposed method achieves accurate registration for remote sensing images with significant resolution and regional variations, and the registration effect is superior to that of other representative comparison methods. However, due to the scarcity of pre- and post-damage image pairs caused by war, the proposed method is unable to conduct more extensive experiments. Therefore, the robustness of the method proposed in this article for multi scenario applications such as heavy occlusion and low-quality image pairs needs further verification, which is also a weakness of the proposed method in this paper.

## 5. Conclusions

This paper proposes a spatial point feature-based registration method for remote sensing images with large regional variations. This method builds descriptors based on the geometrical distribution (distance and orientation) of edge keypoints, achieving accurate registration between image pairs with large regional variations. Meanwhile, the proposed method combines line features with point features, increasing the NCM and reducing the registration errors. Additionally, this method establishes an image pyramid and performs feature extraction within the pyramid. Then, feature descriptors are constructed in the rotated image and matching is conducted within the image pyramid, achieving rotation and scale invariance for this method. The experimental results show that in test images with different scales, rotation angles, and regional differences, the proposed method achieved pixel-level registration root mean square error, and our method’s average registration precision is nearly 100%. Meanwhile, the comparison experiment results show that the proposed algorithm has better matching performance for RS images with resolution and regional variations.

In future work, we will focus on improving the operational efficiency of the algorithm so that this method can be better and faster applied in fields such as disaster assessment and damage effect evaluation. Additionally, we will continuously collect image data to facilitate the deep learning methods’ application in special field.

## Figures and Tables

**Table 1:** Test images’ detailed information.

Group	Image Pairs	Size	GSD (m)	Date	Status
Pre- and Post- attack	#1	720 × 437	0.6	2021	Pre-attack
		716 × 431	0.6	2022	Post-attack
	#2	1024 × 701	0.5	2021	Pre-attack
		960 × 657	0.5	2022	Post-attack
	#3	1024 × 768	1.3	2022	Pre-attack
		1024 × 706	1.3	2022	Post-attack
	#4	1024 × 768	2.0	2023	Pre-attack
		1051 × 801	2.0	1999	Post-attack
Pre- and Post- disaster	#5	1024 × 1024	0.5	2018	Pre-hurricane
		1024 × 1024	0.5	2018	Post-hurricane
	#6	1024 × 1024	0.5	2019	Pre-flood
		1024 × 1024	0.5	2019	Post-flood
	#7	1024 × 1024	0.5	2018	Pre-tsunami
		1024 × 1024	0.5	2018	Post-tsunami
	#8	1024 × 1024	0.5	2018	Pre-wildfire
		1024 × 1024	0.5	2018	Post-wildfire

**Table 2:** Quantitative matching results on Dataset 1.

Status	Indices	Method				
		Method [7]	Method [14]	Method [26]	Method [27]	Ours
Scale_0 & Rot_0	RMSE	6.17	20.00	1.44	1.13	0.96
	NCM	15	1	13	16	24
	Precision	75	13	88	97	100
Scale_2 & Rot_0	RMSE	6.03	20.00	6.23	5.97	1.09
	NCM	10	0	12	7	20
	Precision	75	0	63	65	98
Scale_2 & Rot_45	RMSE	10.77	20	20	15.3	1.05
	NCM	9	0	0	3	16
	Precision	56	0	0	25	98

**Table 3:** Quantitative matching results on Dataset 2.

Status	Indices	Method				
		Method [7]	Method [14]	Method [26]	Method [27]	Ours
Scale_0 & Rot_0	RMSE	6.22	15.41	1.49	1.07	1.02
	NCM	43	9	29	40	57
	Precision	83	25	93	100	100
Scale_2 & Rot_0	RMSE	6.28	20.00	1.74	10.6	1.08
	NCM	29	0	23	16	35
	Precision	75	0	91	63	98
Scale_2 & Rot_45	RMSE	6.18	20	20	10.59	1.07
	NCM	12	0	1	11	31
	Precision	75	0	5	50	98

## Data Availability

The original data presented in the study are openly available in xBD at ”https://xview2.org/dataset” accessed on 5 November 2024.
